# Green synthesis of silver nanoparticles using *Morus nigra* leaf extract and their mechanistic insights into antimicrobial and anticancer effects

**DOI:** 10.3389/fphar.2026.1809089

**Published:** 2026-07-13

**Authors:** Manaal Zahera, Avni Nayyar, Shruti Anand, Shireen Masood, Priya Sharad, Monisha Banerjee, Farzana Mahdi, Lina I. Alnajjar, Mohd Saeed, Nawaf Alshammari, Ahmed M. Alharbi, Archna Talwar

**Affiliations:** 1 Department of Biotechnology, Era University, Lucknow, Uttar Pradesh, India; 2 Department of Chemistry, Isabella Thoburn College, University of Lucknow, Lucknow, Uttar Pradesh, India; 3 Molecular and Human Genetics Laboratory, Department of Zoology, University of Lucknow, Lucknow, Uttar Pradesh, India; 4 Department of Personalized and Molecular Medicine, Era’s Lucknow Medical College and Hospital, Lucknow, Uttar Pradesh, India; 5 Department of Pharmacy Practice, College of Pharmacy, Princess Nourah bint Abdulrahman University, Riyadh, Saudi Arabia; 6 Department of Biology, College of Science, University of Hail, Hail, Saudi Arabia; 7 Department of Medical Laboratory Sciences, College of Applied Medical Sciences, University of Hail, Hail, Saudi Arabia

**Keywords:** Antibacterial, glucose, Morus nigra, MTT assay, silver nanoparticles (Ag NP)

## Abstract

This study reports the preliminary biological evaluation of glucose-capped silver nanoparticles synthesized using an aqueous leaf extract of *Morus nigra*. LC-MS analysis of the methanolic extract provided a preliminary metabolite profile of the plant, confirming the presence of bioactive secondary metabolites. The silver nanoparticles were characterized using various analytical techniques, such as UV-Vis Spectroscopy, FTIR, FESEM, TEM, and DLS/Zeta. 2,2-diphenyl-1-picrylhydrazyl (DPPH) and phosphomolybdate assays demonstrated significantly higher antioxidant potential of GC-AgNPs than aqueous leaf extract. GC-AgNPs reported 57% inhibition (IC_50_ ≈ 65 μg/mL) and a total antioxidant capacity of approximately 446.15 mg AAE/g at 250 μg/mL in the DPPH and phosphomolybdate assays, respectively. The selective antimicrobial dosing against *Escherichia coli* and *Staphylococcus aureus* was determined based on delivery modes. A clear zone of inhibition of 8.33 mm and 12.66 mm against *E. coli* was reported in the disc diffusion method. Contrastingly, *S. aureus* showed inhibition zones of 14 mm and 16 mm in the well diffusion method. The cytotoxic evaluation against SiHa cervical cancer cells using the MTT assay revealed an IC_50_ value of 52 μg/mL for nanoparticles compared to aqueous leaf extract (81 μg/mL). The findings suggest a possible intracellular delivery mechanism; however, this remains hypothetical and requires further validation, including nanoparticle uptake, oxidative-stress pathways, intracellular localization, and others.

## 1 Introduction

Mulberry, belonging to the Morus genus in the Moraceae family, is a rich source of phytochemicals, and the leaves and berries of the mulberry plant are highly valuable (He et al., 2022). Nearly 16 species of the *genus Morus* are found in Africa, Asia, and North America. Among these, the most common species are *Morus nigra*, *Morus alba*, and *Morus rubra*. *Morus nigra* (black mulberry) is a widely cultivated, rustic plant that has been used in traditional Chinese medicine (Pérez-Gregorio et al., 2011). The presence of precious secondary metabolites in the leaves of *M. nigra* possesses pharmacological importance. Studies indicate that black mulberries contain higher levels of total anthocyanins, total phenolics, and total flavonoids compared to other mulberry species (Ercisli and Orhan, 2007). These bioactive compounds have demonstrated various health benefits, including anticancer, antioxidant, anti-diabetic, and neuroprotective effects (Khalifa et al., 2018; Chen et al., 2023).

However, the bioavailability of these bioactive molecules is often limited by poor solubility in physiological fluids and low permeability across biological membranes (Anand et al., 2007). Hence, the incorporation of these phytoconstituents into nanoparticles improves their solubility, stability, and cellular uptake, thereby enhancing their bioactivity (Gunasekaran et al., 2014). Among various metallic nanoparticles, silver nanoparticles are widely studied due to their promising antioxidant, antimicrobial, and anticancer potential (Abass Sofi et al., 2022). The application of green chemistry in the synthesis of silver nanoparticles involves the use of phytochemicals that serve as reducing agents and bind to reduced silver atoms through coordination interactions, resulting in the controlled growth of nanoparticles (Shahzadi et al., 2025). Additionally, the encapsulation of these nanoparticles by a biocompatible moiety stabilizes them by preventing agglomeration and minimizing their surface-area-to-volume ratio (Sidhu et al., 2022). Notably, silver nanoparticles are known for their antimicrobial and anticancer properties through the generation of reactive oxygen species (ROS). The functionalization of these nanoparticles by a capping agent results in a more controlled generation of ROS, thereby tailoring their oxidative and biological efficacy (Verma et al., 2022).

Despite extensive reports on plant-mediated synthesis of nanoparticles, most studies focus on optimizing synthesis parameters and evaluating biological efficacy without establishing a relationship between nanoparticle surface chemistry and efficacy (Chikkamadaiah et al., 2024). In particular, the integrated role of plant-mediated reduction and biocompatible capping agents such as glucose in governing nanoparticle stability, cellular internalization, and bioactivity remains insufficiently understood and is often proposed based on literature-supported hypotheses rather than direct experimental validation. Therefore, there remains a cautious interpretation of structure-activity relationships in green-synthesized nanoparticle systems. Furthermore, recent research focuses on the importance of correlating microstructural features with macroscopic performance, emphasizing that nanoparticle efficacy is strongly influenced by surface interactions, structural evolution, and interfacial properties under physiological conditions. For instance, modelling-based studies have demonstrated that microstructural evolution plays a critical role in determining the mechanical and functional behavior of nanostructured systems (Long et al., 2023). However, such modelling-based approaches are seldom applied in green synthesis systems, where the complexity of plant extracts often limits structure-function analysis.

Although glucose-associated and plant-mediated nanoparticle systems have shown promising biological activity in previous reports, establishing definitive relationships between surface composition, physicochemical stability, and biological performance remains challenging due to the complexity of extract-mediated synthesis systems and limited mechanistic validation. In this context, the present study aims to bridge this gap by integrating metabolic profiling of leaf extract with the controlled synthesis of glucose-capped silver nanoparticles, and their subsequent bioactivity evaluation, with a focus on understanding how surface functionalization influences nanoparticle stability, cellular interaction, and biological activity. The LC-MS analysis provided a preliminary metabolite profile of the leaf extract, revealing the bioactive compounds capable of participating in the green synthesis of nanoparticles. These nanoparticles were analysed for their antimicrobial properties against *Escherichia coli* and *Staphylococcus aureus* using the disc diffusion and well diffusion methods to address the differences in nanoparticle diffusion rates and cell wall permeability. The cytotoxic potential of glucose-capped silver nanoparticles was evaluated using the MTT assay against SiHa cervical cancer cells to examine the effect of glucose capping on nanoparticle internalization and intracellular mitochondrial disruption. This study hypothesizes that surface functionalization was treated as the primary independent variable, while nanoparticle physicochemical properties and biological activity were considered as dependent variables. These measurable endpoints were selected to evaluate the influence of surface modification on the nanoparticle performance. Therefore, these findings represent a preliminary study supporting a Trojan-horse-like mechanism, where surface modification facilitates cellular entry and promotes the intracellular release of bioactive silver ions. However, discussion associated with nanoparticle uptake, intracellular interactions, and ROS-associated effects should be considered a literature-supported hypothetical interpretation, requiring further mechanistic investigation.

## 2 Materials and methods

### 2.1 Chemicals

Silver nitrate (99% purity) [HiMedia Laboratories Pvt. Ltd (Mumbai, India)], D-glucose (anhydrous, 99% purity) [Qualigens (Thermo Fisher Scientific India Pvt. Ltd., Mumbai, India)], 2,2-Diphenyl-1-Picrylhydrazyl (DPPH) (95% purity, SRL, India), sulfuric acid (HiMedia Laboratories Pvt. Ltd., Mumbai, India), sodium phosphate (99% purity, SRL, India), ascorbic acid (99.7% purity, SRL, India), and ammonium molybdate (98% purity, SRL, India).

### 2.2 Sample collection

The leaves of *M. nigra* were gathered from the garden of Isabella Thoburn College (26.8563°N, 80.9499°E) and verified by the herbarium at the National Botanical Research Institute (NBRI), situated in Lucknow, Uttar Pradesh, India.

### 2.3 Preparation of aqueous and methanol leaf extract

The leaves were rinsed with distilled water and dried to eliminate moisture. 15 g of dried leaves were soaked in 200 mL of Milli-Q water and heated in a water bath at 60 °C for 30 min. After cooling, the mixture was left to stand overnight to allow for complete extraction. The resulting solution was filtered using Whatman filter paper No. 1 and kept refrigerated at 4 °C for the synthesis of nanoparticles.

5 g of the powdered leaves were soaked in 75 mL of methanol and allowed to stand for 48 h at room temperature, followed by shaking to facilitate efficient extraction of polar phytochemicals. The filtrate was concentrated by evaporating the solvent in a hot air oven at 45 °C-50 °C. The dried methanolic extract was stored at 4 °C for LC-MS analysis.

### 2.4 Biogenic synthesis of glucose-capped silver nanoparticles (GC-AgNPs)

4 mL sample of *M. nigra* aqueous leaf extract was combined with 36 mL of a 1 mM AgNO_3_ solution and stirred magnetically at 70 °C for one hour. Following this, 0.0072 g of glucose was introduced into the reaction mixture and stirred for an additional hour. The mixture was then centrifuged thrice at 5000 rpm for 10 min, and the resulting pellets were rinsed with Milli-Q water to remove impurities.

## 3 Characterization of glucose-capped silver nanoparticles (GC-AgNPs)

The absorption spectrum of the mixture was measured between 200 and 800 nm using a Shimadzu 1601 spectrophotometer (India). The particle size distribution and surface charge of GC-AgNPs were analyzed with a Malvern Zeta Sizer (United Kingdom). FESEM analysis was conducted using a JEOL JSM 7610f instrument (Japan) to examine the morphology of the GC-AgNPs at an accelerating voltage of 5 kV. TEM imaging was performed using a TECNAI 20G2 from Thermo Fisher (United States). The elemental composition of GC-AgNPs was identified using an Energy Dispersive X-Ray Diffraction spectrum. FT-IR spectra of the sample extract and glucose-capped AgNPs were recorded in the 4000-400 cm−^1^ range using a Bruker Alpha II FTIR Spectrometer (USA). The data were processed using Bruker OPUS software, version 7.5.18.

### 3.1 Liquid Chromatography-Mass Spectrometry (LC-MS) characterization

The chemical constituents of the methanol leaf extract were identified using LC-MS. LC-MS analysis was carried out using a Waters Alliance e2695 equipped with a quaternary, low-pressure mixing pump. The HPLC was interfaced with an HPLC-TQD Mass spectrometer with LC-ESI/MS(LC-MS)2ChScan. Full-scan mode from m/z 150 to 2000 was performed with a source temperature of 150 °C. Separation was achieved using an ACCUCORE C18 column (150 × 2.1 mm, 2.6 µm), with methanol containing 0.1% formic acid as the mobile phase. A gradient elution was applied at a flow rate of 1 mL/min. The MS spectra were recorded in both positive and negative ion modes. The temperature of the drying gas (N_2_) was maintained at 350 °C, with a flow rate of 950 L/h. The nebulizing pressure (N_2_) was adjusted to 21.75 psi. 25 mg of the sample was dissolved in 1 mL of methanol and filtered through a 0.22 μm nylon filter. 2 μL of the extract was injected onto the analytical column for analysis. and transferred to LC vials for analysis.

### 3.2 Antioxidant analysis

#### 3.2.1 DPPH assay

The *in vitro* antioxidant activity was performed using the DPPH method (Qubtia et al., 2024). 1 mL of 0.1 mM DPPH solution prepared in methanol was added to 1 mL of GC-AgNPs at different concentrations (50, 100, 150, 200, and 250 μg/mL). 1 mg/mL of ascorbic acid was used as a positive control. The solutions were incubated in the dark for 30 min, and the absorbance was measured at 517 nm by a UV- Vis spectrophotometer. The % inhibition of GC-AgNPs and the standard was measured using the formula:

$$\%\;Inhibition=\frac{Absorbance\;of\;Control-Absorbance\;of\;Sample}{Absorbance\;of\;Control}$$

#### 3.2.2 Phosphomolybdate assay

The total antioxidant capacity of GC-AgNPs and the *M. nigra* aqueous leaf extract was analysed using the phosphomolybdate assay (Khatoon et al., 2013). 3 mL of the reagent solution containing 0.6 M sulfuric acid, 4 mM ammonium molybdate, and 28 mM sodium phosphate was mixed with test samples of different concentrations ranging from 50, 100, 150, 200, and 250 μg/mL. The sealed test tubes were incubated at 95 °C for 90 min and allowed to cool at room temperature. The absorbance was recorded at 695 nm, with 0.3 mL methanol serving as the blank. The ascorbic acid (AA) was used as a positive control. The total antioxidant activity was expressed as ascorbic acid equivalents (AAE) per gram of sample, calculated from a standard calibration curve of AA.

### 3.3 Antimicrobial activity

The Antibacterial activity was determined by the Zone Inhibition Method (Kirby-Bauer method). The MHA plates were inoculated by spreading 100 µL of bacterial cultures, *E. coli* (MTCC-452) and *S. aureus* (MTCC-96). Inoculum was prepared by adjusting to 0.5 McFarland Unit - approximately cell density (1.5 × 10^8^ CFU/mL) in Mueller-Hinton Broth, followed by placing the discs containing 5 µL of different concentrations (0%–100%). One disc in each plate was loaded with solvent alone, serving as a vehicle control, and a Ciprofloxacin disc (8 µg) was taken as a positive control. The *E. coli* and *S. aureus* plates were incubated at 37 °C for 24 h. A clear zone created around the disc was measured and recorded.

Additionally, the antibacterial activity of *S aureus* was also performed using the well diffusion method. In this method, wells of different concentrations (0–100 µg) were made, and 10 µg of the sample was taken and serially diluted to achieve the required amount to be loaded into the well. One well in each plate was loaded with solvent alone, serving as a vehicle control, and a Ciprofloxacin well was taken as a positive control. The plates of *S. aureus* were incubated (Basil Scientific Corp. India) at 37 °C for 24 h. A clear zone created around the well was measured and recorded.

### 3.4 Cell line maintenance

For cell culture studies, cervical cancer cell line (SiHa) and normal epithelial cell line (NIH/3T3) were used, and the cell lines were procured from the National Centre for Cell Sciences (NCCS), Pune, India. Both the cell lines were cultured and sustained in Dulbecco Modified Eagle Medium (DMEM) enriched with 10% Fetal Bovine Serum (FBS) and 1% antibiotic antimycotic (Ab/Am) solution, maintained until 70˗85% confluency is achieved. The cells were subjected to trypsinization using 0.25% trypsin (Gibco, ThermoScientific), followed by the removal of DMEM medium from the cell culture flask, and incubated for 5 min in 5% (v/v) CO_2_ to facilitate cell detachment and sub-culture.

#### 3.4.1 Cell cytotoxicity assay

Cells (SiHa and NIH/3T3) were grown at a density of 1 × 10^^4^ cells per well in 96-well plates and permitted to adhere overnight. After 24 h, cells were incubated in medium alone (untreated control), vehicle control (DMSO in medium, given at a concentration of 0.1%, equivalent to leaf extract and GC-AgNPs treated groups), positive control (cisplatin, treated at 12 µM), and variable concentrations of leaf extract and GC-AgNPs (5-450 μg/mL). The plate was then placed in a CO_2_ incubator for an additional 24 h. After incubation, 10 µL of MTT solution (5 mg/mL) was added to each well and incubated for 3 h. After that, the supernatant was discarded, and 100 µL of DMSO was added to solubilize the purple-hued formazan crystals. Absorbance was quantified at a wavelength of 540 nm by using a microplate reader (BioTek, USA), and the viability percentage of cells was assessed (Sachan et al., 2025). To evaluate selective cytotoxic effects, a non-cancerous epithelial cell line, NIH/3T3, was used. Statistical analysis was performed utilizing GraphPad Prism software. The following formula was used to calculate percent cell viability:

$$\%\;cell\;viability=\frac{Absorbance\;of\;treated\;cells}{Absorbance\;of\;untreated\;cells}\times 100$$

#### 3.4.2 DCFDA analysis for ROS quantification

To validate the cytotoxic mechanism, the intracellular ROS generated was quantified using 2′,7′-Dichlorofluorescin Diacetate (DCFDA) dye. SiHa cells were cultured in 12-well plates. After 24 h, cells were treated with 52 μg/mL of aqueous leaf extract and GC-AgNPs, and 200 µM of H_2_O_2_ was used to account for positive controls in cancer cell line. After 24 h, treatment media was removed and cells were washed with 1X PBS. In each well, 10 μM DCFDA was added and incubated in dark at 37 °C for 30 min. The cells were again washed with 1X PBS and examined using fluorescence microscope (Nikon Eclipse Ts2, Japan) with a 200× magnification (Sharad et al., 2026).

### 3.5 Statistical Analysis

All statistical analyses were conducted using OriginPro 2024b and GraphPad Prism software. For the MTT assay, three independent experiments were performed in triplicate, and IC_50_ (inhibitory concentration) and CC_50_ (cytotoxic concentration) values were calculated using a non-linear regression method. The percentage cell viability was fitted to a four-parameter logistic (4 PL) model (log [inhibitor] vs. response-variable slope). For the DCFDA assay, three independent experiments were performed in duplicates, and statistical analysis was performed using one-way analysis of variance (ANOVA) followed by Tukey’s *post hoc* test. Statistical significance was considered at a threshold of p < 0.05. The graphs were plotted using Microsoft Excel, OriginPro 2024b, and GraphPad Prism.

## 4 Results and discussion

Although the glucose-capped silver nanoparticles were synthesized using an aqueous leaf extract, the LC-MS profiling of the methanolic extract of *M. nigra* reveals a comprehensive list of metabolites that are likely to be present in the aqueous extract. Methanol is known to extract a wider range of bioactive compounds, including both polar and moderately non-polar metabolites. It is important to note that the phytochemical profile of the aqueous leaf extract may not fully correspond to that of the methanolic extract. However, key classes of metabolites contain functional groups such as -OH, -NH_2_, and -COOH that facilitate metal ion reduction and nanoparticle stabilization, thereby supporting the role of *M. nigra* aqueous leaf extract as an effective bioreductant in nanoparticle synthesis (Adeyemi et al., 2022). The proposed mechanism of green synthesis of silver nanoparticles involves the complexation of Ag^+^ ions with phytochemicals, resulting in the nucleation of Ag^0^. Subsequently, glucose adsorbs onto the nanoparticle surface, forming a hydrophilic steric barrier that enhances colloidal stability (Anand et al., 2025). Therefore, the synergistic role of the phytochemical reducing agent and glucose capping results in stable and biocompatible nanoparticles, as demonstrated in our earlier studies (Talwar et al., 2025).

However, the reaction time employed in the experiment was greater than that used in other literature sources for the green synthesis of silver nanoparticles. The increase in time duration was used to ensure that the total reduction of silver ions depends on the composition and concentration of phytochemicals present in the extract (Okka et al., 2023). Furthermore, glucose primarily functions as a capping and stabilizing agent, potentially enhancing nanoparticle stability and biological interactions. Polydispersity in nanoparticle sizes may be attributed to several aspects of plant-mediated systems of nanoparticle synthesis. Firstly, heterogeneous nucleation occurs due to the existence of numerous phytochemical compounds in the extract, which act as reducing agents at different rates, leading to the simultaneous formation of nuclei at different rates (Deka et al., 2025). Secondly, the variation in phytochemical-mediated reduction kinetics by various metabolites possessing varying redox potentials leads to the uneven growth of nanoparticles (Khan et al., 2022). In addition, the prolonged reaction time could facilitate additional growth mechanisms such as Ostwald ripening, whereby small particles dissolve and redeposit on larger particles, thus increasing the range of particle sizes (Cardoso et al., 2025). Overall, these parameters account for the production of polydisperse nanoparticle systems, a phenomenon commonly observed in biosynthesis using complex biological extracts. Furthermore, systematic optimization of synthesis parameters, including reaction time and precursor concentrations, was not extensively performed in this study, which represents a limitation and requires investigations on optimizing these conditions to achieve better control over nanoparticle size distribution and uniformity.

While the present study demonstrates the synthesis and preliminary biological evaluation of glucose-capped silver nanoparticles, certain limitations, such as a lack of *in vivo* validation and detailed toxicity profiling, must be acknowledged, and parameters such as the scalability of synthesis must be explored in future investigations.

### 4.1 Phytochemical profiling

#### 4.1.1 Liquid Chromatography-Mass Spectrometry

**FIGURE 1:**
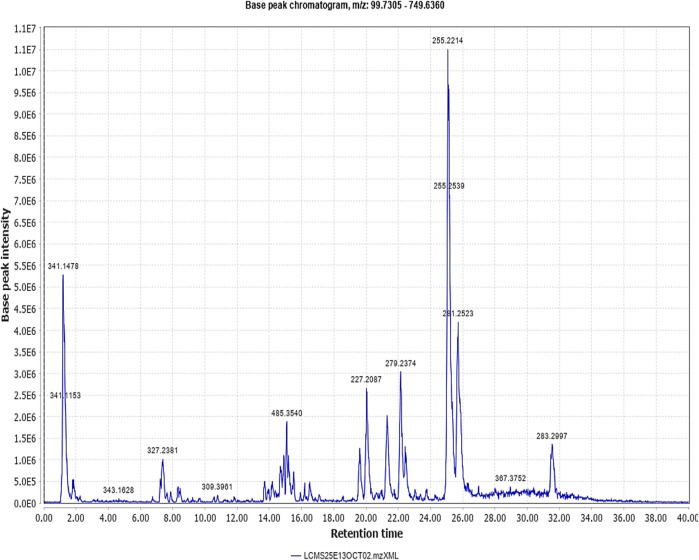
Total ion chromatogram of the negative ion mode of the methanolic leaf extract of *Morus nigra*.

**FIGURE 2:**
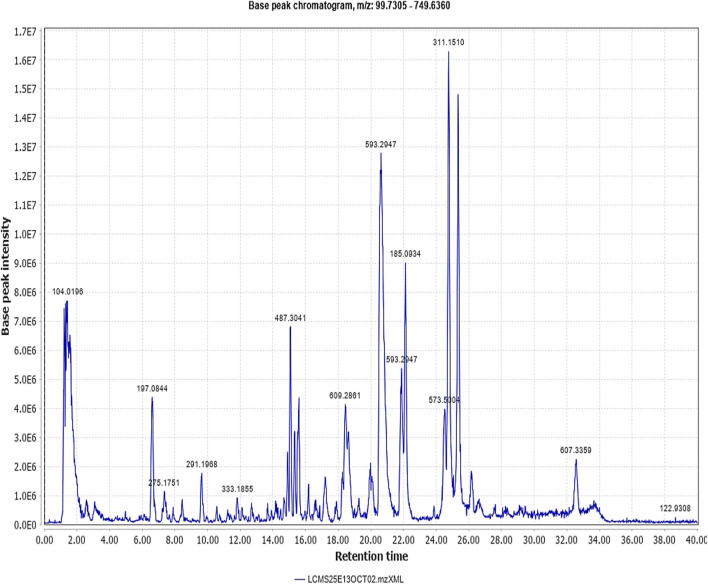
Total ion chromatogram of the positive ion mode of the methanolic leaf extract of *Morus nigra.*

The phytochemical profiling of the methanolic extract of *M. nigra* was performed using Liquid Chromatography-Mass Spectrometry (LC-MS), focusing on key compounds identified by their retention times, molecular formulas, and spectral matching scores obtained from the MassBank database. In the negative ion mode, organic acids such as (S)-malate (m/z 133.01) and 2-oxoglutarate (m/z 145.01), and phenolic acids (arbutin and ellagic acid) were identified. However, identification of geniposide (an iridoid glycoside), uncommon in the Moraceae family, was observed at RT 23.75 min and should be considered a tentative assignment based on LC-MS data (Figure 1; Table 1). Six major metabolites, including amino acids (methionine), organic acids (2-oxoglutarate), small fatty-acid derivatives, and non-protein amino acid candidates such as azetidine-2-carboxylate, were annotated in the positive ion mode. A characteristic ellagic acid fragment was also detected at RT 22.12 min. The identification of genipin (an iridoid-type aglycone) at RT 20.63 min requires further structural validation (Figure 2; Table 1).

**TABLE 1:** Tentative compounds identified from the methanolic leaf extract of *Morus nigra* leaves by LC-MS.

Retention time (min)	Tentative compound	Molecular formula	Ion type	Precursor m/z	Scores	Phytochemical class
7.39	Pentanoate	C_5_H_10_O_2_	[M−H]^-^	101	0.984	Fatty acid
13.69	(S)-Malate	C_4_H_6_O_5_	[M−H]^-^	133.01	0.791	Organic acid
14.20	L-homoserine	C_4_H_9_NO_3_	[M−H]^-^	118.05	0.7551	Amino acid derivative
14.89	Arbutin	C_12_H_16_O_7_	[M−H]^-^	271.08	0.4563	Phenolic glycoside
15.19	2-Oxoglutarate	C_5_H_6_O_5_	[M−H]^-^	145.01	0.9535	Organic acid
20.03	Ellagic acid	C_14_H_6_O_8_	[M−H]^-^	301	0.3757	Polyphenol
23.75	Geniposide	C_17_H_24_O_10_	[M−H]^-^	387.12	0.8069	Iridoid glycoside
6.60	Methionine	C_5_H_11_NO_2_S	[M + H]^+^	148.20	0.9849	Amino acid
14.90	3-Methylbutanoic acid	C_5_H_10_O_2_	[M + H]^+^	101	0.7222	Fatty acid derivative
20.63	Genipin	C_11_H_14_O_5_	[M + H]^+^	225.07	0.8452	Iridoid type metabolite
22.12	Ellagic acid	C_14_H_6_O_8_	[M + H]^+^	299.15	0.3151	Phenolic compound
22.77	2-Oxoglutarate	C_5_H_6_O_5_	[M + H]^+^	145.01	0.9694	Organic acid
24.53	Azetidine-2-carboxylate	C_4_H_7_NO_2_	[M + H]^+^	100	0.9093	Non-protein amino acid

### 4.2 UV spectroscopy

**FIGURE 3:**
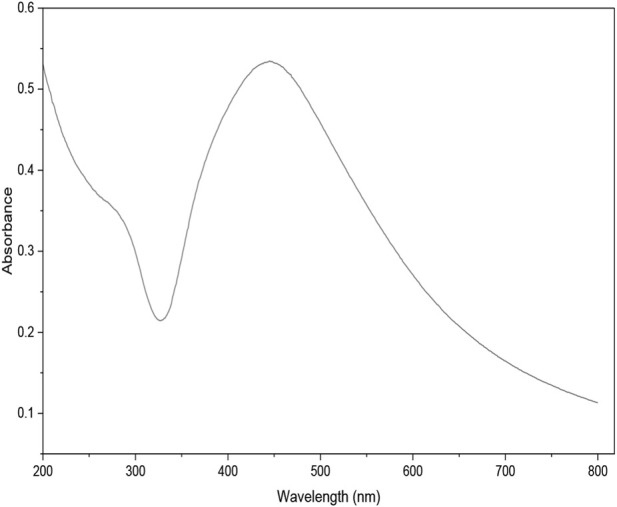
UV-Vis absorption peak of GC-AgNPs.

The biogenic synthesis of silver nanoparticles was carried out using the aqueous leaf extract of *Morus nigra*, resulting in the reduction of Ag^+^ to Ag^0^. The addition of plant extract to AgNO_3_ resulted in a change in color to brownish-red, attributed to surface plasmon resonance (SPR), which is indicative of the formation of silver nanoparticles (De Mel et al., 2025). The spectrophotometric analysis of the green-synthesized GC-AgNPs showed maximum absorption at 440 nm (Figure 3). The FWHM was found to be 49.6 nm, suggesting a relatively improved size distribution of nanoparticles with reduced agglomeration compared to highly broadened peaks.

The UV-Vis spectra of the synthesized nanoparticles exhibited a relatively broad surface plasmon resonance (SPR) peak, which may be due to variations in particle size, shape anisotropy, and interparticle interactions, leading to damping of plasmon oscillations (Debu et al., 2017). Additionally, the presence of surface-bound phytochemicals and glucose capping may influence the local environment around the nanoparticles, contributing to peak broadening (Kelly et al., 2003).

### 4.3 SEM/EDX analysis

**FIGURE 4:**
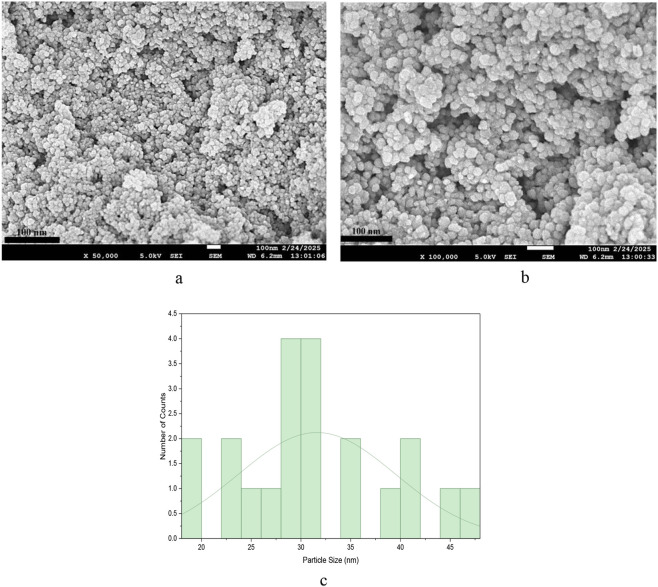
FESEM images of GC-AgNPs **(a)** X50,000, **(b)** X,100,000, **(c)** Nanoparticle size Histogram GC-AgNPs [avg 31 nm].

The morphology of the synthesized nanoparticles was analyzed using scanning electron microscopy (SEM) at different magnifications (Figures 4a,b). The micrographs obtained show that the particles are spherical with little agglomeration (Shankar et al., 2017). The particle size distribution ranges from 18 to 47 nm, with an average size of 31 nm (Figure 4c).

**FIGURE 5:**
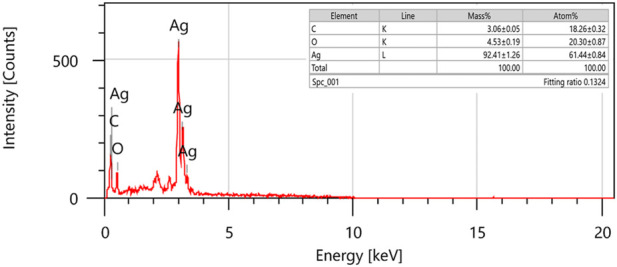
Energy dispersive X-Ray diffraction spectrum of GC-AgNPs.

The EDX spectrum demonstrated the elemental composition profile of the silver nanoparticles. The most intense peak at nearly 3 keV confirms the presence of metallic silver (Gharari et al., 2023). The EDX spectrum showed absorption peaks corresponding to carbon and oxygen, confirming the presence of an organic capping agent (glucose) (Figure 5).

### 4.4 TEM/SAED analysis

**FIGURE 6:**
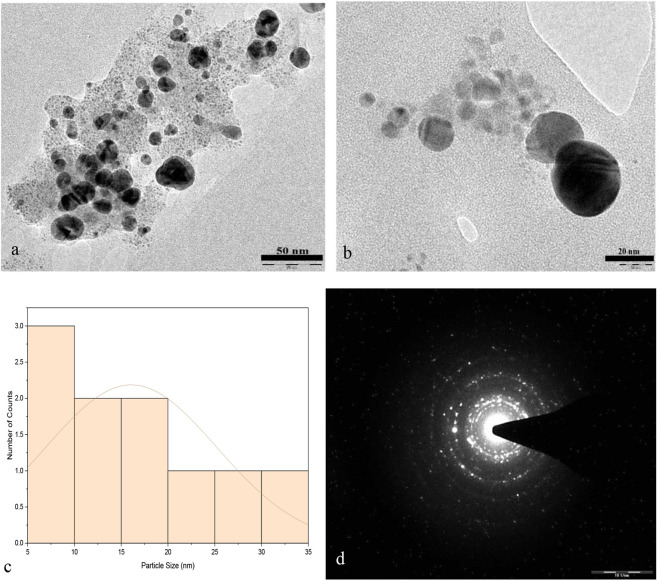
TEM micrographs of GC-AgNPs **(a)** 50 nm, **(b)** 20 nm, **(c)** Nanoparticle Size Histogram [avg 16 nm], **(d)** SAED of GC-AgNPs.

TEM micrographs further reveal spherical particles with an average size of 16 nm (Figures 6a–c). However, the polydispersity observed in TEM micrographs correlates with the broad SPR peak observed in UV-Vis spectra, supporting the presence of nanoparticles with variable sizes and possible agglomeration. Furthermore, little agglomeration was observed, which may be due to interparticle interactions or incomplete surface passivation, commonly reported in biogenically synthesized nanoparticles (Sidhu et al., 2022).

The crystalline nature of the biosynthesized nanoparticles was confirmed by the bright concentric circles observed in the selected area electron diffraction pattern (SAED) (Figure 6d) (Alampally et al., 2025). These diffraction rings correspond to the characteristic lattice planes of silver arising from the diffraction of electrons by randomly oriented nanocrystals, indicating a polycrystalline structure consistent with the face-centered cubic (fcc) arrangement of metallic silver (Sun and Xia, 2002). Therefore, the observed crystallinity further supports the successful reduction of silver ions into well-defined metallic nanoparticles.

### 4.5 Dynamic light scattering/zeta potential

**FIGURE 7:**
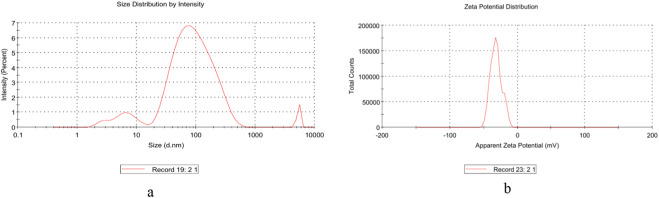
**(a)** Size distribution of GC-AgNPs **(b)** Zeta potential of GC-AgNPs.

A Dynamic Light Scattering experiment assessed the size distribution of biogenically synthesized GC-AgNPs. The DLS technique relies on the scattering intensity of particles, which inherently biases it toward measuring larger particle sizes (Al-Khafaji et al., 2020; Wilson and Prud’homme, 2021). The Z-average particle size was observed at 103 nm (Figure 7a), which is larger than the size reported by FESEM and TEM analysis. The polydispersity index (PDI) of GC-AgNPs was reported at 0.283. The stability of the nanoparticles is evaluated by the surface charge of the nanoparticles, determined by zeta potential measurements. The zeta potential of the synthesized nanoparticles was recorded at −31.4 mV (Figure 7b). Zeta potential values greater than ±30 mV are considered stable due to sufficient electrostatic repulsion between particles (Bhattacharjee, 2016). The observed negative charge suggests the presence of surface-bound biomolecules, contributing to surface functionalization. The negative charge on the nanoparticles induces repulsive forces, which prevent nano-agglomeration and maintain their stability (Das et al., 2019). Moreover, high surface charge may also enhance the interaction of these glucose-capped nanoparticles with biological systems, including cellular uptake and protein adsorption.

However, there was an inconsistency among the results obtained using different methods of characterization, which can be attributed to the fundamental differences in measurement principles. TEM and FESEM methods are used to determine the actual physical size of the nanoparticles in a dried state, measuring their metallic cores without the influence of surrounding solvent layers (Williams and Carter, 1996). In contrast, dynamic light scattering (DLS) measures the Z-average size of nanoparticles in colloidal suspension, which includes not only the metallic core but also the surface-bound phytochemicals, glucose capping layer, and associated hydration shell (Filippov et al., 2023). Additionally, DLS measurements are sensitive to nanoparticle aggregation and Brownian motion, which can further increase the apparent particle size (Filippov et al., 2023). Therefore, the larger particle size observed in DLS compared to TEM and FESEM is expected and reflects the combined effects of solvation, surface functionalization, and possible aggregation in the colloidal system.

### 4.6 FT-IR spectroscopy

**FIGURE 8:**
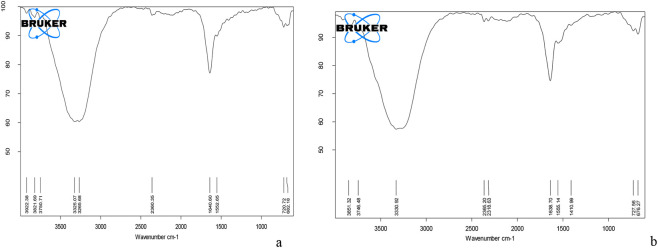
FT-IR spectrum **(a)** Aqueous Leaf extract of *Morus nigra* **(b)** FT-IR spectrum of GC-AgNPs.

The investigation into the functional groups of aqueous leaf extract responsible for reducing silver ions is conducted using FT-IR spectroscopy. The complex nature of aqueous leaf extract is confirmed by absorption peaks, i.e., 3922, 3821, 3750, 3325, 3265, 2360, 1640, 1552, 720, 682 cm^-1^ (Figure 8a). The peaks of aqueous leaf extract were compared with the silver nanoparticles, which display peaks at 3851, 3746, 3330, 2365, 2318, 1638, 1410, 727, and 676 cm^-1^ (Figure 8b). The bands in the 2790-3570 cm^-1^ region are associated with the stretching vibration of C-H and O-H bands (Walkowiak-Bródka et al., 2022). The 2000-2400 cm^-1^ region corresponds to the stretching of C≡C and C≡N bonds. The peaks in the 1630-1690 cm^-1^ region represent the C=O stretching of carbonyl groups or N-H bending of primary amides (Melkamu and Bitew, 2021; Pungle et al., 2022). The bands at 1500–2000 cm^-1^ confirm the presence of a benzene ring (Buchweitz et al., 2012). The band at 1410 cm^-1^ observed in the spectrum of silver nanoparticles corresponds to the C-H bending vibration of glucose (Louise, n.d.). The bands in the range of 600-800 cm^-1^ could be attributed to alkene halogens or alkenes (Pungle et al., 2022). Therefore, the presence of identified phytochemicals in the synthesized nanoparticles provides evidence for the similarity in the spectral pattern of aqueous leaf extract and GC-AgNPs.

### 4.7 Antioxidant analysis

#### 4.7.1 DPPH assay

**FIGURE 9:**
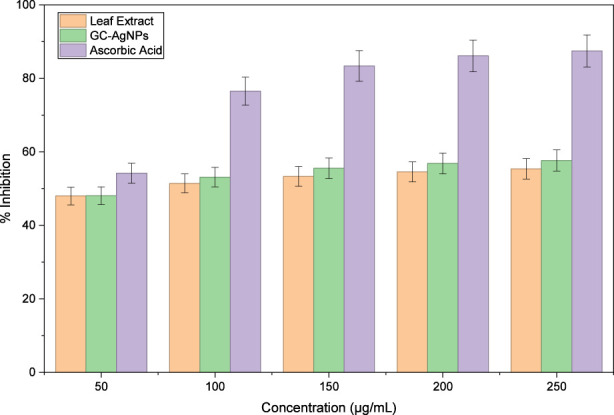
DPPH Assay of *Morus nigra* Aqueous Leaf Extract, GC-AgNPs, and Ascorbic Acid, showing concentration-dependent potential.

GC- AgNPs and *Morus nigra* demonstrated antioxidant activity, as shown in Figure 9, in a concentration-dependent manner. GC-AgNPs showed a maximum of 57% inhibition at 250 μg/mL (IC_50_ = 65.08 ± 0.06), while the aqueous leaf extract exhibited 55% inhibition (IC_50_ = 74.27 ± 0.08) at the same concentration (Table 2). However, the percentage scavenging activity of ascorbic acid was comparatively higher, 87.4% at 250 μg/mL (IC_50_ = 44.96 ± 0.0). These results show that coating nanoparticles with a biocompatible molecule may lead to a synergistic interaction between the silver core, glucose layer, and residual phytochemicals. This enhances their interaction with DPPH radicals compared to dispersed phytocomponents in the aqueous leaf extract, highlighting the role of nanoparticles in antioxidant-based therapeutic applications.

**TABLE 2:** Total antioxidant activity (TAC) and DPPH Assay of GC-AgNPs and the aqueous leaf extract.

S.No.	Samples	TAC (mg AAE/g)	IC_50_ (µg/mL)
1	GC-AgNPs	446.15 ± 2.11 mg AAE/g	65.08 ± 0.06 μg/mL
2	Aqueous Leaf extract	263.4 ± 1.97 mg AAE/g	74.27 ± 0.08 μg/mL

#### 4.7.2 Phosphomolybdate assay

**FIGURE 10:**
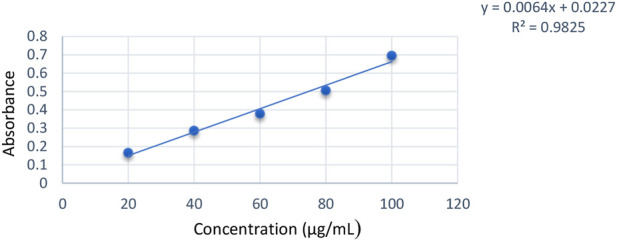
The total antioxidant capacity was determined using a standard calibration curve of ascorbic acid. The calibration curve exhibited good linearity, with a line equation of y = 0.0064x + 0.0227 and a correlation coefficient (*R* ^2^ = 0.9825).

The total antioxidant capacity (TAC) of GC-AgNPs and *Morus nigra* aqueous leaf extract was determined using a calibration curve and expressed as milligrams of ascorbic acid equivalents (mg AAE/g). TAC of GC-AgNPs and aqueous leaf extract was reported at 446.15 ± 2.11 mg AAE/g and 263.4 ± 1.97 mg AAE/g, respectively (Figure 10, Table 2). These findings suggest that small-sized GC-AgNPs exhibit better colloidal stability than aqueous leaf extract due to the greater accumulation of reducing equivalents in the formulated nanoparticles, resulting in a more effective reduction of the phosphomolybdate reagent by silver nanoparticles.

### 4.8 Antibacterial analysis

**FIGURE 11:**
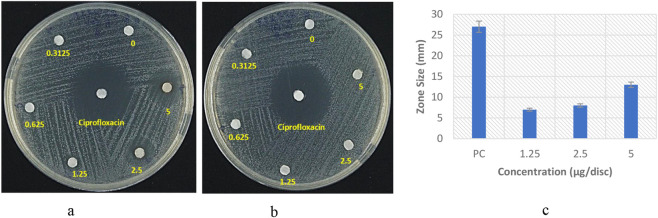
Antibacterial activity of GC-AgNPs and Aqueous Leaf Extract against *Escherichia coli* using the disc diffusion method: **(a)** GC-AgNPs, **(b)** Aqueous Leaf Extract, **(c)** Zone of Inhibition at varying concentrations of GC-AgNPs against *Escherichia coli*; Ciprofloxacin was used as the positive control.

**FIGURE 12:**
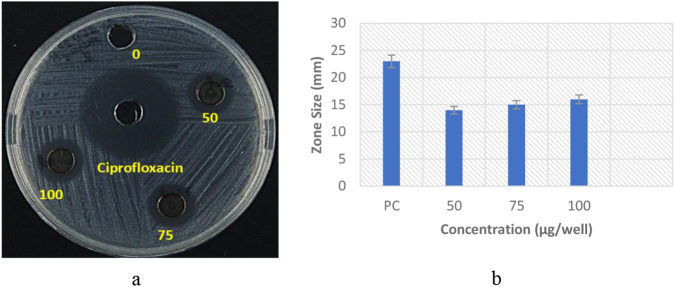
Antibacterial activity of **(a)** GC-AgNPs against *Staphylococcus aureus* by well diffusion method; **(b)** Zone of Inhibition at varying concentrations of GC-AgNPs against *Staphylococcus aureus*; Ciprofloxacin- positive control.

The preliminary antibacterial activity of GC-AgNPs was determined using the disc diffusion method against Gram-negative *Escherichia coli*, Gram-positive *Staphylococcus aureus* bacteria, and aqueous leaf extract. The diameter of the inhibition zone demonstrated the inhibitory activity of silver nanoparticles. Notably, no zone of inhibition was observed in the aqueous leaf extract, more likely due to the lower concentration of the extract (Figure 11b). A clear zone of inhibition against *E. coli* was reported at 8.33 ± 0.57 mm and 13 ± 0.57 mm at concentrations of 2.5 μg/disc and 5 μg/disc, respectively (Figures 11a,c). No zone of inhibition was observed for *S. aureus* under identical conditions. However, a clear zone of inhibition at 14 ± 1.00 mm (50 µg/well) and 16 ± 1.15 mm (100 µg/well) was observed for *S. aureus* in the well diffusion method (Figures 12a,b). This is due to the differential diffusion kinetics between these methods. The well diffusion method allows a more uniform and sustained release of nanoparticles into the bacterial lawns, while the disc diffusion method facilitates diffusion of nanoparticles outwards from a fixed point source, thereby decreasing the concentration gradient over distance (Kourmouli et al., 2018; Chung et al., 2023). Furthermore, the differential response of these bacteria can be attributed to the structural differences in their cell walls. The cell wall of *E. coli* is porous and possesses a thin peptidoglycan layer, which may allow easy diffusion of nanoparticles. Conversely, the thick peptidoglycan layer of *S. aureus* can act as a physical barrier, requiring a higher concentration of nanoparticles to reach bactericidal levels (Hochvaldová et al., 2024). In this context, the antibacterial activity of GC-AgNPs may involve a Trojan-horse-like pathway, enabling nanoparticle uptake at higher local concentrations across the bacterial cell envelope, followed by intracellular release of bioactive silver ions (Santiago-Tirado et al., 2017). This delayed intracellular ion release results in effective bacterial growth inhibition, even in the presence of a thick peptidoglycan layer that acts as a diffusion barrier (Santiago-Tirado et al., 2017). Therefore, this study demonstrates that surface functionalization of silver nanoparticles creates a hydrophilic and negatively charged shell that can influence their colloidal properties (Mikhailova, 2020). This, in turn, may regulate the differential release kinetics of Ag^+^ ions based on mode of delivery methods (disc diffusion vs. well diffusion) and affect the interaction of glucose-capped nanoparticles with Gram-positive and Gram-negative bacteria, highlighting the importance of selective antimicrobial dosing against different bacterial strains.

This study employs different antimicrobial assay methods (disc diffusion and well diffusion) to account for differences in nanoparticle diffusion behavior and interactions with bacterial cell walls. While these methods provide qualitative and semi-qualitative insights into antimicrobial activity, more standardized approaches, such as determining the minimum inhibitory concentration (MIC) and minimum bactericidal concentration (MBC), would enable a more precise evaluation of antibacterial efficacy.

### 4.9 Cell cytotoxicity assay

**FIGURE 13:**
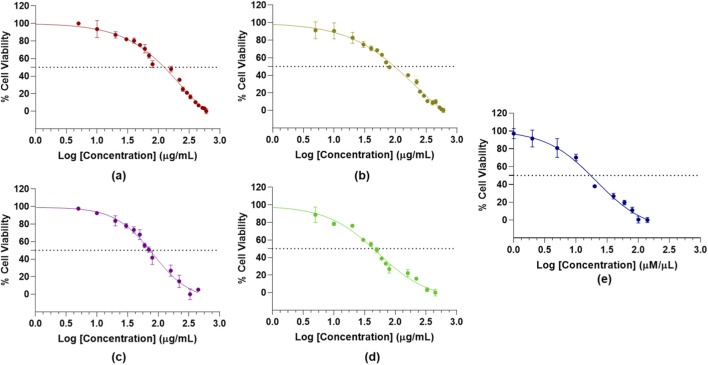
Dose-response curve generated for NIH/3T3 cell line with the treatment of **(a)** aqueous leaf extract and **(b)** GC-AgNPs and SiHa cell line with the treatment of **(c)** aqueous leaf extract, **(d)** GC-AgNPs, and **(e)** cisplatin (positive control). Data derived from three independent experiments in triplicate.

The anti-cervical cancer potential of aqueous leaf extract and GC-AgNPs was evaluated by assessing their cytotoxicity against adherent human cervical cancer cells (SiHa cells) using the MTT assay. The MTT experiment for both aqueous leaf extract and GC-AgNPs markedly reduced the viability of human cervical cancer cells. The results indicated that the viability of cancer cells significantly decreased compared to the control cells (untreated cells) in a dose-dependent manner (Figure 13). Furthermore, the selective targeting was also checked in normal cells (NIH/3T3), and it was found that the aqueous leaf extract and GC-AgNPs demonstrated high cytotoxic concentrations (CC_50_). Additionally, the selectivity index demonstrated that GC-AgNPs were better at targeting cancer cells as compared to Aqueous plant extract (Table 3).

**TABLE 3:** IC_50_ and CC_50_ in cancerous and non-cancerous cell lines and their selectivity index.

​	NIH/3T3		SiHa		​
Treatment	CC_50_ (95% CI)	*R* ^2^	IC_50_ (95% CI)	*R* ^2^	Selectivity index
Aqueous plant extract	230.6 (163.7–417.2)	0.982	81.32 (69.23–103.3)	0.978	2.835
GC AgNPs	196.9 (132.1–413.7)	0.981	51.60 (41.92–71.38)	0.973	3.815
Cisplatin	—	—	21.07 (15.47–35.75)	0.977	-

**FIGURE 14:**
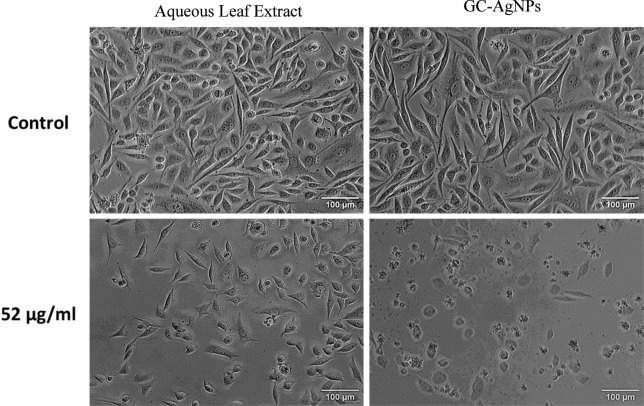
Morphological analysis of SiHa cells with treatment of aqueous leaf extract and GC-AgNPs at 52 μg/mL concentration by using phase contrast microscopy.

IC_50_ values determined using a dose-response curve for aqueous leaf extract and GC-AgNPs were found to be around 81.32 μg/mL (69.23–103.3) and 51.60 (41.92–71.38) µg/mL, respectively, after 24 h of treatment (Table 3). The results were further supported by cellular morphology analysis post-24-h treatment, which demonstrated increased cell death and loss of viability with the treatment at 52 μg/mL of GC-AgNPs compared to the aqueous leaf extract (Figure 14). The lower IC_50_ of GC-AgNPs suggests enhanced anti-cervical cancer properties relative to aqueous leaf extract.

#### 4.9.1 DCFDA assay for ROS

**FIGURE 15:**
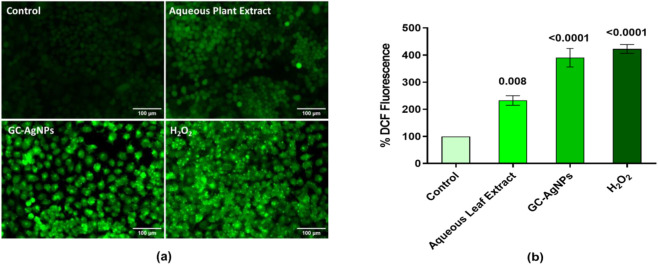
Intracellular ROS determination by DCFDA assay. **(a)** Fluorescence microscopic images of SiHa cells treated with 52 μg/mL of aqueous leaf extract and GC-AgNPs, and 200 µM of H_2_O_2_ (positive control) along with the untreated (control) group. **(b)** Quantification of ROS generation (% DCFDA fluorescence) in both treated and untreated SiHa cells. Values are expressed as a percentage, where 100% represents control. Data are expressed as the mean ± SD of three independent experiments.

Both the aqueous leaf extract and GC-AgNPs induced oxidative stress in SiHa cells. Increased green fluorescence intensity in GC-AgNPs-treated cells indicated that more ROS production occurred as compared to aqueous leaf extract, whereas the control group showed minimal fluorescence intensity (Figure 15). The treatment of H_2_O_2_ served as a positive control for ROS generation analysis. The increased ROS level in GC-AgNPs-treated cells indicates that oxidative stress may be an important mechanism to promote anticancer activity. High quantities of ROS can damage to cellular biomolecules, leading to cell death.

The surface functionalization of silver nanoparticles with glucose may facilitate their preferential uptake by cancer cells, suggesting a possible Trojan-horse-mediated intracellular delivery mechanism. This process is hypothesized to promote nanoparticle internalization through nutrient-mimicking pathways rather than direct membrane disruption (Zhang et al., 2016; Morais et al., 2022). Moreover, the controlled intracellular release of silver ions induces oxidative stress and mitochondrial dysfunction, leading to delayed morphological deterioration (Géczi et al., 2023; Zheng et al., 2024). This effect is evidenced by reduced MTT activity, excess ROS generation, and cell death-like morphological changes, indicating that cellular damage predominantly arises after intracellular accumulation of silver nanoparticles (Morais et al., 2022). However, direct experimental validation of the molecular mechanism, cellular uptake pathways, intracellular release of Ag^+^ ions, long-term effects, apoptosis-marker analysis, or pathway-level validation was not performed in this study. Therefore, the proposed mechanism should be considered as a hypothesis requiring further investigation. In contrast to uncapped silver nanoparticles, where silver ion exposure primarily occurs extracellularly, glucose-capped nanoparticles enable intracellular ion release, thereby minimizing non-specific toxicity and enhancing mechanistic selectivity.

## 5 Conclusion

This study reports the biogenic synthesis of glucose-capped silver nanoparticles using an aqueous leaf extract of *Morus nigra*, integrating metabolic profiling, physicochemical characterization, and biological assays within a single framework. LC–MS analysis of the methanolic extract provided preliminary phytochemical insight into the plant source; however, direct correlation with the aqueous extract used for nanoparticle synthesis requires further validation. Furthermore, glucose capping effectively modulated the surface properties of the nanoparticles, enhancing their biocompatibility for potential therapeutic applications. The glucose-capped silver nanoparticles exhibited improved antioxidant activity compared to the aqueous leaf extract, as confirmed by the DPPH assay and phosphomolybdate assay. Furthermore, the silver nanoparticles demonstrated delivery-mode-dependent antibacterial efficacy against *E. coli* and *S. aureus* under different diffusion assays. In addition, glucose-capped silver nanoparticles showed a lower IC_50_ against SiHa cervical cancer cells compared to the aqueous leaf extract, accompanied by apoptosis-like morphological changes. However, several limitations, such as tentative metabolite identification based on LC-MS analysis, were tentative and performed using a methanolic extract, which may not completely represent the phytochemical composition of the aqueous leaf extract. The antimicrobial assessment without determination of minimum inhibitory concentration (MIC) or minimum bactericidal concentration (MBC) limits quantitative comparison. This study also lacks validation of the mechanistic pathway involved in cytotoxicity. Nonetheless, the proposed Trojan-horse-like mechanism remains hypothetical and is supported by indirect biological evidence; direct confirmation of intracellular uptake and silver ion release kinetics requires further investigation. Despite these limitations, this work highlights the therapeutic potential of *M. nigra* derived glucose-capped silver nanoparticles, and future studies should focus on their comprehensive biological evaluation to establish their therapeutic applicability.

## Data Availability

The raw data supporting the conclusions of this article will be made available by the authors, without undue reservation.
